# Blood-brain barrier dysfunction in disorders of the developing brain

**DOI:** 10.3389/fnins.2015.00040

**Published:** 2015-02-17

**Authors:** Raffaella Moretti, Julien Pansiot, Donatella Bettati, Nathalie Strazielle, Jean-François Ghersi-Egea, Giuseppe Damante, Bobbi Fleiss, Luigi Titomanlio, Pierre Gressens

**Affiliations:** ^1^INSERM U1141, Robert Debre's HospitalParis, France; ^2^Université Paris Diderot, Sorbonne Paris Cité, UMRS 1141-PROTECTParis, France; ^3^PremUPParis, France; ^4^S. Maria della Misericordia Hospital, Università degli Studi di UdineUdine, Italy; ^5^Lyon Neurosciences Research Center, INSERM U1028, CNRS UMR5292 – Lyon UniversityLyon, France; ^6^Brain-iLyon, France; ^7^Department of Division of Imaging Sciences and Biomedical Engineering, Centre for the Developing Brain, St. Thomas' HospitalLondon, UK; ^8^Pediatric Emergency Department, APHP, Robert Debré HospitalParis, France

**Keywords:** WMD, TBI, BBB, stroke, hypoxia-ischemia, brain, developemental desorders

## Abstract

Disorders of the developing brain represent a major health problem. The neurological manifestations of brain lesions can range from severe clinical deficits to more subtle neurological signs or behavioral problems and learning disabilities, which often become evident many years after the initial damage. These long-term sequelae are due at least in part to central nervous system immaturity at the time of the insult. The blood-brain barrier (BBB) protects the brain and maintains homeostasis. BBB alterations are observed during both acute and chronic brain insults. After an insult, excitatory amino acid neurotransmitters are released, causing reactive oxygen species (ROS)-dependent changes in BBB permeability that allow immune cells to enter and stimulate an inflammatory response. The cytokines, chemokines and other molecules released as well as peripheral and local immune cells can activate an inflammatory cascade in the brain, leading to secondary neurodegeneration that can continue for months or even years and finally contribute to post-insult neuronal deficits. The role of the BBB in perinatal disorders is poorly understood. The inflammatory response, which can be either acute (e.g., perinatal stroke, traumatic brain injury) or chronic (e.g., perinatal infectious diseases) actively modulates the pathophysiological processes underlying brain injury. We present an overview of current knowledge about BBB dysfunction in the developing brain during acute and chronic insults, along with clinical and experimental data.

## Introduction

The blood-brain barrier (BBB) is a physical barrier essential for the maintenance of a precisely regulated intracerebral microenvironment. Its characteristics limit paracellular diffusion while allowing the tightly controlled receptor-mediated endocytosis of larger molecules and the transporter-mediated intake of smaller nutrients like glucose, insulin and iron. Endothelial cells interact closely with other central nervous system (CNS) cells such as neurons, pericytes and astrocytes, through adherens junctions, influx and efflux transporters, metabolic enzymes, the extracellular matrix, astrocytic endfeet etc. All these structures together constitute the Neurovascular Unit (NVU), and are believed to be essential for the regulation of BBB permeability (Ballabh et al., 2004).

For a long time, the BBB was considered “incomplete” during fetal life. However, there is ample evidence to suggest that the BBB is already well developed (Ballabh et al., 2005). For instance, the formation of tight junctions (TJs) takes place at the same time as angiogenesis (Virgintino et al., 2004), and even the first vessels to invade the avascular neuroectoderm are impermeable to albumin and immunoglobulins. Nevertheless, fetal brain vessels exhibit different, and in some respects increased, transport properties for amino acids and other metabolites, reflecting the higher demand for nutrients in the developing CNS (Ek et al., 2001, 2006; Johansson et al., 2006, 2008; Saunders et al., 2012). Understanding the changes that occur in the BBB during normal development is critical, as it is not only a site of possible vulnerability to injury but also a potential therapeutic route for injuries occurring during the perinatal period. Perinatal brain damage is one of the leading causes of lifelong disability, including cerebral palsy, seizure disorders, sensory impairment, and cognitive limitations (Low, 2004). This brain damage could be due to inflammatory, hypoxic-ischemic, hemorrhagic or excitotoxic mechanisms, or a combination of these. The lifetime cost of the care of one child affected by cerebral palsy is about 1 million US dollars (CDC CfDCaP, 2004), highlighting the need to develop new strategies for treatment and prevention. The only treatment with proven efficacy for perinatal brain injury is hypothermia, but this can only be applied safely to term infants with hypoxic-ischemic encephalopathy. Hypothermia in these infants reduces brain injury, doubling the chances of survival without clinical deficits (Edwards et al., 2010). However, more efforts are needed to find novel neuroprotective treatments for perinatal brain damage, especially in preterm infants. Designing neuroprotective molecules requires a detailed knowledge of the pathophysiology of brain damage, including BBB damage, in this population. This will facilitate the delivery of neuroprotective molecules, whose efficacy is strictly dependent on their capacity to cross brain barriers.

Currently, translational research is increasingly focused on the broader functional aspects of the brain response to injury, shifting from cell-oriented studies to experimental research on physiological concepts such as BBB integrity. This is due to the fact that dysfunctions of the BBB, such as the impairment of TJ formation/function, are a contributing factor in a number of neurological diseases in adults and infants. In models of perinatal injuries in term infants, cerebrovascular endothelial cells can be damaged by hypoxic-ischemic insults, an activated excitotoxic cascade or traumatic brain injury, leading to impaired BBB function. The link between BBB dysfunction and injury due to the dysregulation of developmental processes, such as occurs to a greater extent in preterm infants, is less well studied.

The aim of this review is to describe current understanding of BBB dysfunction in the developing brain during the most common subtypes of injury, supported by clinical and experimental data.

### Basics of BBB development and age-related differences in function

The specific barrier characteristics of BBB endothelial cells within the developing CNS are induced during angiogenesis by complex crosstalk with cellular and acellular elements. During development, the CNS is vascularized by the angiogenic sprouting of vascular networks from the surrounding mesoderm in a precise spatiotemporal manner that differs among species. Which cell type is responsible for BBB differentiation has not yet been clarified: astrocytes have long been considered as the main source of BBB-inducing signals, but barrier induction most likely takes place well before astrocyte differentiation, so there is a probable influence of neuroblast cells or pericytes (Bauer and Bauer, 2000; Armulik et al., 2010; Daneman et al., 2010a). These different structures and cells have different maturation rates across species and across developmental stages, so animal models are not always representative of the human situation in every respect.

With regard to the developmental changes in the BBB, the presence of TJs in cerebral blood vessels and the expression of influx and efflux transporters is even higher at mid-gestation than in adulthood; for instance, TJs appear as early as 8 weeks of gestation in humans, at 13 days in mice and at postnatal day (P) 5 in *Monodelphis* opossums, which is when the first vessels appear in the neocortex (Ek et al., 2012). These TJ proteins are functional, as recent ultrastructural studies have demonstrated that the TJs of both endothelial cells in cerebral blood vessels and choroidal epithelial cells in embryos and neonates restrict the passage of low-molecular-weight molecules (Ek et al., 2003, 2006; Johansson et al., 2006). However, as these blood vessels do not display the properties of mature vessels with respect to pericyte coverage or junctional organization, it is probable that their BBB properties are not yet fully mature. This has been confirmed in part by a recent study showing that several hundreds of genes are differentially expressed between early postnatal and adult brain endothelial cells, indicating differences in molecular and probably physiological properties (Daneman et al., 2010b). However, the implication of these differences in the BBB across developmental stages for brain health remains unknown (Engelhardt and Liebner, 2014).

## Principal insults to the developing brain and their animal models

### Germinal matrix hemorrhage

**Figure 1:**
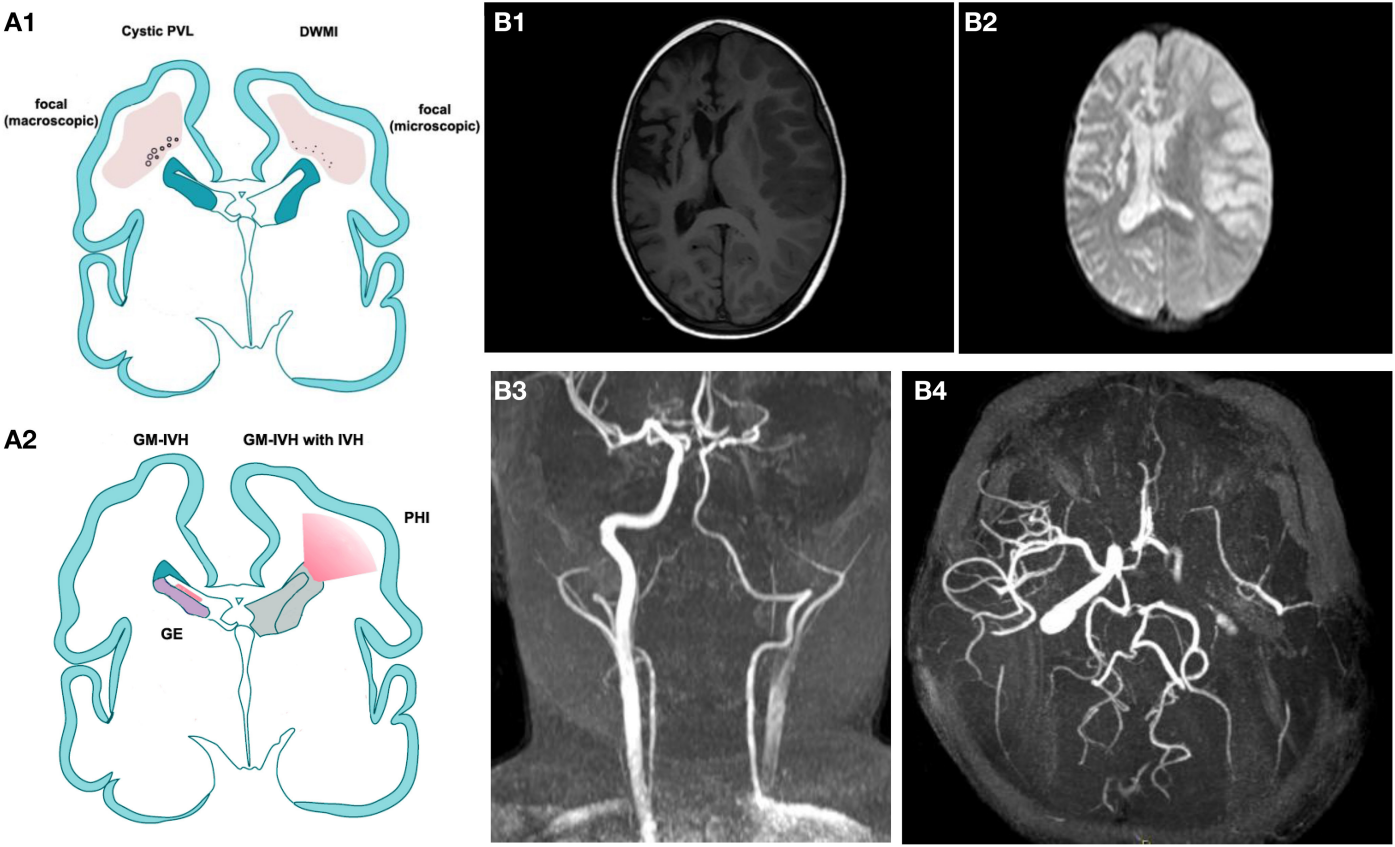
**(A)** Modified by J. Volpe, Lancet Neurology 2009. **(A1)** Cystic Periventricular Leucomalacia (PVL) and Diffuse White Matter Injury (DWMI) and **(A2)** germinal matrix haemorrage-intraventricular Haemorrage (GMH-IVH) and GMH-IVH with periventricular Haemorrage infarction (PHI). Schematic coronal sections from the brain of a 28-week-old premature infant. Color key: Focal necrotic lesions in cystic PVL (small circles), focal necrotic lesions in DWMI (black dots), diffuse cellular changes in both cystic PVL and DWMI (pink), hemorrhage into ganglionic eminence (red) that results in IVH in the ependymal (left) or PHI (right). **(B,B1)** T1 weighted image illustrating an acute right ischemic stroke is a sickle cell disease children previously affected by a left ischemic stroke. **(B2)** Brain diffusion-weighted imaging (DWI) sequence of the same patient. **(B3,B4)** a 3D time-of-flight magnetic resonance angiography (MRA) suggesting dissection of the left internal carotid (**B2**: coronal view, **B3** transverse view).

Intraventricular hemorrhage of the germinal matrix (GM-IVH) causes very substantial and permanent injury, and is the leading cause of hydrocephalus in children. GM-IVH occurs when a hemorrhage in the GM breaks through the ependyma and into the lateral ventricles (see Figure 1). About 12,000 premature infants develop IVH every year in the United States alone (Vohr et al., 1999; Ballabh, 2010), with the incidence in extremely premature infants being approximately 45% (Wilson-Costello et al., 2005). Infants with a history of IVH have a higher incidence of seizures, neurodevelopmental delays, cerebral palsy and death. The incidence of IVH in very low birth weight infants (<1500 g) declined from 40% in the early 1980s to 20% in the late 1980s (Philip et al., 1989). However, because of the sharply increased survival of extremely premature infants, in the last two decades, the incidence of IVH has remained stable (Jain et al., 2009). Thus, IVH continues to be one of the major problems faced by premature infants in modern neonatal intensive care units worldwide. The etiopathogenesis of GM hemorrhage is multifactorial, with a combination of vascular and intravascular factors considered to be responsible. It is necessary to understand the reason for the vulnerability of GM microvessels to hemorrhage in order to develop therapeutic strategies.

GM-IVH has been modeled in several animal species including the dog, rabbit, sheep, rat, mouse and pig, either by directly injecting blood into the ventricles or by changing hemodynamic properties, such as blood pressure, the volume of blood circulating, serum glycerol, carbon dioxide, osmolarity or oxygenation levels (Goddard et al., 1980; Yoshioka et al., 1989; Balasubramaniam and Del Bigio, 2006; Georgiadis et al., 2008; Tosun et al., 2013). The two most-used animals models of IVH are the intracerebral injection of sterile collagenase, a hemorrhage-inducing agent (Krafft et al., 2012; Lekic et al., 2012), in mice, and the intracerebroventricular injection of fresh homologs blood in adult Sprague-Dawley rats or in piglets at 9–22 h of life (Aquilina et al., 2007, 2012). The appropriate model is selected based on the pathophysiology of hemorrhage induction and injury progression. The blood injection model mimics a rapidly progressing hemorrhage, whereas collagenase enzymatically disrupts the basal lamina of brain capillaries, causing an active bleed that generally evolves over several hours. The effects of GM-IVH on the BBB have not been extensively assessed in any of these models.

### White matter damage

White matter damage (WMD) is a characteristic injury of preterm infants, involving destructive microcystic lesions within the white matter, the disrupted maturation of oligodendrocytes and hypomyelination (Degos et al., 2010a) (see Figure 1). Although WMD is also observed in term infants, the relative contributions of direct injury to oligodendrocytes/myelin and secondary injury due to gray matter damage are still being defined. WMD is a healthcare priority, as it is the main cause of neurological handicap in preterm infants and the principal cause of cerebral palsy (Tahraoui et al., 2001). On a pathophysiological level, based on strong clinical evidence and a wealth of studies in animal models, the hypothesis of a purely hypoxic-ischemic cause of injury has been replaced by a multifactorial hypothesis. The central role of inflammation is now appreciated, given its contribution in terms of excess cytokines, free radicals, increased excitatory amino acid release (excitotoxicity), and trophic factor imbalance (Lipton and Rosenberg, 1994; Evrard et al., 1997; Yoon et al., 1997; Back et al., 1998). Clinical spectroscopy data do support a role for hypoxia-ischemia in the pathophysiology of WMD, but it is increasingly evident that inflammation-induced endothelial cell dysfunction and changes in glial metabolism may contribute substantially to these effects (Schuhmann et al., 2003; Lodygensky et al., 2014). The multifactorial hypothesis includes the concept of sensitization, whereby vulnerability to hypoxia-ischemia or excitotoxicity is enhanced by the preexistence of inflammation (Hagberg and Mallard, 2005; Favrais et al., 2007, 2011), which in the clinical setting is reported to occur in at least 40% of preterm infants.

Experimental paradigms that have addressed the pathophysiology of WMD include models in the mouse, rat and sheep using inflammation, excitotoxicity and hypoxia-ischemia (Tahraoui et al., 2001) (see Table 1). A well-characterized murine model of perinatal excitotoxic-induced WMD consists of the i.c. injection of the glutamate analog ibotenate, a potent neurotoxin that activates the N-methyl-D-aspartate receptor, in 5-day-old mouse pups (Marret et al., 1995). Ibotenate is commonly used to induce brain lesions in both the hippocampus and the white matter, in which it stimulates these receptors present on oligodendrocytes and microglia (Degos et al., 2010b), leading to microcysts similar to those seen on post-mortem examination in preterm infants (Verney et al., 2012). In this model, vulnerability to WMD is increased by systemic inflammation (Dommergues et al., 2003; Favrais et al., 2007; Aden et al., 2010), and the BBB is slightly affected.

**Table 1:** **Some of the principal animal models mentioned in this review with correlate references**.

**Human pathologic condition**	**Animal used and age of the animal**	**Type of damage “inflicted”**	**References**
GMH-IVH	Rabbit 29 day-gestation	Glycerol administration 3 h post-delivery	Ballabh et al., 2005
	Dog (terme beagle pups)	Phenilephrine hydrocloride i.v. injection	Baud et al., 2004
	Sprague-Dawley Rat P7	Intracerebral injection of glibenclamide	Bauer and Bauer, 2000
	Pregnant Wistar Dam	Intrauterine injection of glibenclamide	Bednarek et al., 2012
	CD-1 mice	Intrastriatal double injection of autologous whole blood, Intrastriatal injection of bacterial collagenase	Biran et al., 2008
	Piglet	i.c.v. injection of fresh homologs blood	Bolton et al., 1998; Blond et al., 2002
Diffuse white matter injury	Cat pregnant	LPS injections	Briones et al., 2011
	Rabbit pregnant	*E. coli* injections	Briones et al., 2011
	Dog (beagle) P14	Bilateral carotid occlusion	Briones et al., 2011
	Swiss Mouse P5	Interacerebral ibotenate injections	Briones et al., 2011
	Long Evans Mouse P7	Unilateral carotid ligation+ hypoxia	
H-I	P7 Wistar rats	Permanent occlusion of the Middle Cerebral Artery (MCA), pMCAo + O2 8%	Evrard et al., 1997
	Rice's model adapted to other rat's ages	“	Favrais et al., 2007, 2011
	Mouse P7	Blockade of the past External Carotid Artery-Internal Carotid Artery (ECA-ICA) bifurcation	Feng et al., 2008
		External-internal carotid artery	Favrais et al., 2011
	Mouse P8	Permanent occlusion of the Middle Cerebral Artery (MCA), pMCAo + O2 8%	Feng et al., 2008
	Mouse P10	“	Feng et al., 2008
	Mouse P12	“	Feng et al., 2008
Stroke	Wistar Rat P7	pMCAo +bilateral transient Common Carotid Artery (tCCAo) occlusion	Feng et al., 2008
	Wistar Rat P10	“	Feng et al., 2008
	CB17 mouse P12	pMCAO	Favrais et al., 2011
TBI			
Fluid percussion injury (FPI) models			
Middle	Rat	Fluid-percussion brain injury of graded severity	Labus et al., 2014
	Cat Rabbit	Fluid-percussion injury (3.5 atm.) Pressure wave produced by a fluid-percussion device	Labus et al., 2014
	Sheep Dog		Labus et al., 2014
Lateral	Rat	Fluid-percussion brain injury of graded severity	Labus et al., 2014
	C57BL/6 Mouse	Right-sided parasagittal FPI	Labus et al., 2014
	Newborn pig	Lateral FPI moderate severity (1.9–2.1 atm)	Labus et al., 2014
Weight-drop models	Rat	Impact injuries in the hindpaw cortical area segmented brass weight free-falling through a Plexiglas guide tube free-falling weight with a final impact of 0.01 J	Labus et al., 2014
	Rat adult		Labus et al., 2014
	C57BL/6 Mouse (10–16 weeks)		Labus et al., 2014
Controlled cortical impact injury Models	Ferret	stroke-constrained pneumatic impactor varying magnitudes of controlled cortical impact	Labus et al., 2014
	Mouse	pneumatic impactor	Labus et al., 2014
	Rat	CCI of velocity 3.5 m/s and dwell time 400 ms CCI + hypothermia	Labus et al., 2014
	Yorkshire Swine		Labus et al., 2014
	Monkey		Labus et al., 2014
	Cat	Cerebral wound made with a steel sphere with a velocity of 240–300 m/s (0.9–1.4 J)	Labus et al., 2014
Penetrating Ballistic-like injury model	Sprague-Dawley rats	Simulated ballistic wound to the right frontal hemisphere implemented by an inflatable penetrating probe	Labus et al., 2014
	C57Bl/6 Mouse pregnant (15 days of gestation)	LPS intrauterine injections	Labus et al., 2014

Animal models of preterm brain injury have also conclusively shown that exposure to inflammation alone, even at low levels, is sufficient to induce WMD similar to that seen clinically (Rousset et al., 2006; Bednarek et al., 2012). In addition to WMD, the exposure of fetal sheep to low-dose endotoxin leads to increased BBB permeability, as assessed by staining for plasma albumin extravasation (Yan et al., 2004). This effect has been noted in the cortical white and gray matter and the thalamus, but not the cerebellum. WMD comparable to that seen in preterm infants can also be induced in the mouse or rat brain by the Vannucci hypoxia-ischemia method at P3 or P5 or by chronic exposure to hypoxia (P3–P11) (Hagberg et al., 2002). There is very little information regarding the BBB in these models of preterm WMD.

### Hypoxic-ischemic encephalopathy

Hypoxic-ischemic encephalopathy (HIE) is considered a major cause of brain injury in term neonates. At present, there are no specific serum biomarkers or other tools to easily assess neurological injury at birth, either in terms of its severity or the prediction of neurodevelopmental outcome. Because of this knowledge gap, all newborns with moderate or severe encephalopathy are treated with hypothermia in an identical fashion. Randomized controlled trials of therapeutic hypothermia have demonstrated a reduction in death or severe disability at 18 months, but death and disability continue to occur in 30–70% of infants with severe to moderate HIE despite cooling therapy (Shankaran et al., 2005; Azzopardi et al., 2009; Tagin et al., 2012). The criteria used to identify neonates with so-called HIE are (1) a pH ≤ 7 or base deficit ≥16 mEq/L in umbilical cord arterial plasma, and (2) a history of an acute perinatal event or non-availability of arterial blood gas or a pH or base deficit in the borderline range associated with a 10-min APGAR score ≤5 or assisted ventilation at birth. An understanding of the neuropathological process that leads to HIE could lead to the identification of new markers of neuronal injury that correlate with disease and neurodevelopmental outcome, and thus facilitate a more targeted therapeutic approach using adjunctive therapies (Chalak et al., 2014).

To study the effects of oxygen and nutrient deprivation on the developing brain, the species most commonly used are sheep, rabbits, piglets and, most of all, rodents. Differences in temporal phases of development between human and animal brain structures make the choice of a specific species and the determination of a precise correlation between developmental stages in the animal model and the corresponding stage in neonatal brain development very complex. Based on several experimental studies evaluating the developmental profile of the main neural cells of interest, the scientific community has established that a reasonable comparison can be drawn between P3/5 rodents and preterm infants, and P7/10 rodents and term infants (Kinney and Volpe, 2012).

Across the wide range of species used to model HIE, the relative contribution of vessels supplying blood to the brain is variable (Purves, 1972). In rodents, brain ischemia induced by the occlusion of a single carotid artery is not always consistent, and injury is typically induced by combining this with systemic hypotension. This combination of insults in the P7 rat, known as the Rice-Vannucci model, was originally developed in 1981 and is considered the pioneering model of HIE (Rice et al., 1981). It has since been adapted to many postnatal ages and transferred to the mouse (Derugin et al., 1998; Sheldon et al., 1998; Ohshima et al., 2012; Tsuji et al., 2013). The brain's response to global hypoxia-ischemia is a multistep process. Within the first few hours, regionally specific increases of cerebral blood flow occur followed by decreases due to regional vasoconstriction or the collapse of cardiac output, with the subsequent onset of excitotoxicity, energy depletion and the generation of free radicals, leading to apoptotic and necrotic cell death and the generation of edema (Ferrari et al., 2010a). This phase is followed within a few hours/days by a second phase consisting of a neuroinflammatory response, mitochondrial permeabilization, reperfusion and loss of cerebral autoregulation, and the production of free radicals (Hamrick and Ferriero, 2003; McLean and Ferriero, 2004). Recent hypotheses describe a tertiary phase that occurs days or even years after the injury and that contributes to the persistence of inflammation and reduced efficacy of brain repair (Fleiss and Gressens, 2012).

The effects of hypoxia-ischemia on the adult BBB include the disruption of TJ proteins (in particular claudin-5 (Cld5), occludin (Occl), zonula occludens (ZO) -1 and -2 (Bolton et al., 1998; Fischer et al., 2002; Mark and Davis, 2002; Witt et al., 2003; Sandoval and Witt, 2008; Zehendner et al., 2013) and increased BBB permeability in *in vitro* studies (Fischer et al., 2000; Zhang et al., 2000; Mark and Davis, 2002). These events are mediated by proinflammatory cytokines such as tumor necrosis factor (TNF) α, interleukin (IL)-1β, IL-8, monocyte chemoattractant protein-1, intercellular adhesion molecule-1, vascular endothelial growth factor (VEGF) and nitric oxide (NO), and astrocytes seems to play a protective role (for a review, see Ballabh et al., 2004). However, differences between the mature and developing brain limit the possibility of extrapolating data from adult studies to neonates.

Concerning developmental age, there are few comparative studies between barriers in the adult and developing brain in this specific model, and the results are not always concordant; in fact, studies in sheep show that the BBB becomes less susceptible to hyperosmolar stress with age (Stonestreet et al., 2006), and in rodents, it seems to be more vulnerable to hypoxia-ischemia at early stages of development (Muramatsu et al., 1997). Studies in rodent models of neonatal hypoxia-ischemia have indicated that the integrity of brain barriers is compromised (Muramatsu et al., 1997; Ferrari et al., 2010b; Tu et al., 2011; Yang et al., 2013a), a finding confirmed by a study of cerebrospinal fluid (CSF) samples from HIE infants showing that the ratio of the albumin concentration in the CSF to that in the plasma is 5 times higher than normal, suggesting leaky brain barriers in these infants (Kumar et al., 2008). Most of these studies, however, precede more recent knowledge of BBB characteristics suggesting that the BBB is more resistant during the developmental period than after maturation (Johansson et al., 2008; Ek et al., 2012; Saunders et al., 2012). Additionally, these early studies did not examine specific TJ disruptions or undertake a qualitative evaluation of the passage of substance and molecules, assessing only immunoglobulin (Ig) G labeling at different time points. Further investigations are needed to better understand BBB behavior in this model.

### Stroke

Ischemic stroke occurs when cerebral blood flow is locally interrupted due to a clot within a vessel (Davis and Donnan, 2012) (see Figure 1). Perinatal stroke is defined as a cerebrovascular event that occurs between 28 weeks of gestation and a postnatal age of 28 days. Its incidence is similar to that in the elderly, approximately 1 in 4000 live births (Lynch et al., 2002), and it is associated with cerebral palsy [which is correlated with infarct size (Lee et al., 2005)], epilepsy, language delays and behavioral abnormalities. Classically associated with hypoxia, recent animal models have used permanent middle cerebral artery occlusion in association with transient bilateral common carotid artery occlusion to mimic an isolated acute ischemic event followed by reperfusion on resuscitation. Currently, more reproducible models are the 1998 Renolleau model in P7 Wistar rats and the 2013 Tsuji model in P9 CB-17 mice (Charriaut-Marlangue et al., 2013). After stroke in adults, BBB leakage/disruption can occur either transiently, in two distinct phases (Belayev et al., 1996; Rosenberg et al., 1998), or continuously (McColl et al., 2008; Kuntz et al., 2014). Although the basic mechanisms of neurodegeneration after stroke are shared across age groups, immaturity critically affects brain susceptibility and response to ischemia-related insults, including modes of neuronal death, inflammation, leukocyte-mediated injury and susceptibility to reactive oxygen species (ROS) (Benjelloun et al., 1999).

### Traumatic brain injury

Traumatic Brain Injury (TBI) in children can cause more severe cognitive and behavioral deficits than comparable injuries in mature brains, and is one of the most common reasons for the development of significant lifelong disability in a child (Anderson et al., 2005; Rivara et al., 2012; Stanley et al., 2012; Roozenbeek et al., 2013). This is particularly important if we consider that children under the age of 4 years more frequently undergo TBI than any other age group (Koepsell et al., 2011) and that injured infants under the age of 12 months are at high risk of requiring intensive care support (Keenan et al., 2003). A similar developmental sensitivity to TBI can be seen in a rodent model of TBI, in which TBI inflicted at P7 causes the maximum damage. In addition, during the first 3 weeks of life, rodents display high sensitivity to excitotoxicity, as seen during the developmental period in human brains (i.e., maximal brain growth, synaptogenesis, and myelination). Thus, P7 is considered the period of maximal sensitivity and the best age to evaluate effects of TBI in rodents (Bittigau et al., 1999).

TBI can be simulated in different ways in various animal models: in the 1980s, a variety of species including cats, dogs, pigs and non-human primates were used (Xiong et al., 2013), but since the 1990s the use of rodents has predominated thanks to the ease with which experiments can be carried out and managed, and the limited costs. In addition, the ability to genetically modify mice has confirmed their position as the most-used species in laboratory research (Longhi et al., 2001; Duhaime, 2006). Models of focal TBI are the most common, induced by the impact of an object on the head (contusion) or the sudden arrest of the head during motion (deceleration) not necessarily requiring contact with an object. The most common modes of contusion trauma induction are lateral fluid percussion injury (Thompson et al., 2005), controlled cortical impact injury (Dixon et al., 1991; Cernak, 2005; Ajao et al., 2012; Pop et al., 2013) and focal TBI through a closed skull (Chen et al., 1996). The primary injury process in TBI is mechanical damage (vascular damage and bleeding caused by shear forces), immediately followed by mast cell degranulation (Stokely and Orr, 2008), and a secondary pathological phase characterized by excitotoxicity, ischemia, mitochondrial dysfunction and apoptosis (Xiong et al., 2013). This phase leads to the activation of inflammatory processes, which are themselves neurotoxic (Hagberg et al., 2012) and mediated by immune cells resident in the brain as well as by the transmigration of systemic immune cells and BBB disruption (Ramlackhansingh et al., 2011).

An electron microscopic analysis of rat cerebral slices in an animal model of TBI (Dietrich et al., 1994) has revealed the presence of microscopic hemorrhagic contusions or petechia, especially in the white-gray matter interface underlying the somatosensory cortex and cisterna ambiens, demonstrating that primary shear-stress-induced vascular damage can be found distant from the initial injury site, indicating protein leakage and the extravasation of blood cells. The choroid plexus is also activated by TBI inflicted at a distance from the ventricles, leading not to blood-cerebrospinal fluid barrier disruption but to the homing of choroidal monocytes and neutrophils and the synthesis and secretion into the CSF of inflammatory chemokines (Szmydynger-Chodobska et al., 2009, 2012).

## Role of the BBB in the pathophysiology of developmental brain insults

It would be difficult to thoroughly analyze in this review the large amount of information available concerning the possible mechanisms that influence BBB disruption in the developing brain. We will therefore focus on a few selected factors.

### Excitotoxicity and free radicals

NO is a weak free radical produced by the action of nitric oxide synthase (NOS). The direct effects of this molecule on the BBB during development have not been studied so far. However, studies in adult rats demonstrate that in a model of stress-induced neurodegeneration, the production of NO and related oxidative-nitrosative compounds via the expression of inducible NOS (iNOS) correlates with BBB disruption, as evaluated by [^14^C]-sucrose uptake by brain tissue. The injection of the specific iNOS inhibitor 1400 W prevents this increase in BBB permeability. Since the developing brain is considered to be extremely vulnerable to free radical damage due to its high lipid content (O'Brien and Sampson, 1965), relatively high oxygen consumption and capacity for ROS generation, and low concentrations of the main antioxidant enzymes (Baud et al., 2004; Vannucci and Hagberg, 2004; Miller et al., 2012), a potential role of this free radical could be suspected.

Following neonatal hypoxia-ischemia, both neuronal (n) NOS and endothelial (e) NOS are up-regulated, but while nNOS knockout mice seem to be protected following neonatal hypoxia-ischemia (Ferriero et al., 1996), studies indicate that eNOS can play a neuroprotective role in the adult brain by influencing neural migration and outgrowth and acting as a downstream regulator of angiogenesis (Huang et al., 1996). Further studies are needed to clarify the different roles of specific NOS isoforms and their link with BBB disruption.

Concerning white matter insults, in the presence of an activated excitotoxic cascade, damage to cerebrovascular endothelial cells should cause alterations in BBB function that could exacerbate neuronal injury and death. Glutamic acid or glutamate the most important excitatory neurotransmitter in the CNS, also acts in the pathophysiology of neuronal damage. Excess glutamate is known to induce neuronal death through a process in which the overactivation of ionotropic and metabotropic glutamate receptors triggers several intracellular signaling pathways, leading to apoptosis, necrosis or both (Gudino-Cabrera et al., 2014). Glutamate agonists, such as ibotenate, are frequently used to reproduce excitotoxic insults in the CNS. However, there are, to our knowledge, no studies that directly evaluate the effects of excitotoxic insults on BBB permeability during development.

The pathophysiology of TBI can also be explained in part by free radical formation and the effects of these on the BBB. During TBI, the transmigration of circulating immune cells and local microglial activation leads to the production of NO, ROS and inflammatory mediators that are capable of interfering with the BBB (Perez-Asensio et al., 2005; Briones et al., 2011). For instance, the ROS-induced peroxidation of polyunsaturated fatty acids in cell membranes gives rise to active aldehydes, significantly increasing endothelial monolayer permeability in experimental studies (Chodobski et al., 2011). In addition, oxidative stress is associated with the endogenous antioxidant glutathione and the increased permeability of the BBB, although only to low-molecular-weight markers (Agarwal and Shukla, 1999), and hydrogen peroxide has been demonstrated to increase BBB permeability via the Extracellular-signal-regulated Kinase (ERK) signal transduction pathway, along with the redistribution of Occl and ZO-1 and -2 (Fischer et al., 2005).

### Alterations of the basal lamina and endothelial cells

One class of enzymes extensively studied for its role in the BBB is family of proteases known as the matrix metalloproteinases (MMPs). The MMPs cleave their protein substrates based on a conserved mechanism that involves the interaction of an active-site-bound water molecule with a Zn^2+^ ion. The MMPs participate not only in pathophysiological processes but also in many normal biological processes such as embryonic (including brain) development, organ morphogenesis, blastocyst implantation, bone remodeling and wound healing (Brew et al., 2000). Because of this dual nature of the MMPs, it would be interesting to uncover their role in the developing brain after neonatal hypoxia-ischemia and, more importantly, their long-term role in neurological function. MMPs disrupt the BBB by degrading TJ and basal lamina proteins, thereby leading to BBB leakage, leukocyte infiltration, brain edema and hemorrhage (Liu et al., 2012). The activity of the MMPs is regulated by a group of endogenous proteins called tissue inhibitors of metalloproteinases (TIMPs), which bind to active and alternative sites of activated MMPs.

MMPs have been implicated in cerebral ischemia, and several studies have revealed that plasma MMP-9 concentrations strongly correlate with stroke severity in patients and that MMP-9 inhibition attenuates early BBB disruption and cerebral edema while promoting the expression of TJ proteins and angiogenesis (Rosenberg et al., 1998; Liu et al., 2009; Gu et al., 2012; Yang et al., 2013b). For instance, in a mouse model of focal cerebral ischemia, the deletion of the TIMP-1 gene results in increased MMP-9 protein expression and gelatinolytic activity, and is accompanied by exacerbated BBB disruption, neuronal apoptosis and ischemic injury when compared to wild-type animals (Fujimoto et al., 2008). Correspondingly, BBB leakage is reduced by TIMP-1 overexpression, and 24 h infarct volumes are also reduced (Maier et al., 2006). MMP-9 has been shown to degrade TJ proteins (Cld-5, Occl, ZO-1) in animal models of focal cerebral ischemia (Yang and Rosenberg, 2011). Aberrant MMP-9 proteolytic activity degrades not only TJ proteins but also basal membrane proteins (e.g., fibronectin, laminin, collagen, and others). This degradation is associated with an increase in BBB permeability, resulting in brain infarction, edema and hypertension in both animal models (Lee et al., 2007; Rosenberg and Yang, 2007; Yang et al., 2007) and human patients (Gasche et al., 2001; Castellanos et al., 2003; Lo, 2008). In a study conducted in P7 rat pups, hypoxia-ischemia was induced by unilateral ligation of the right carotid artery followed by hypoxia, and a broad-spectrum MMP inhibitor was injected intraperitoneally (i.p.). This early MMP inhibition provided both acute and long-term neuroprotection by reducing TJ protein degradation, preserving BBB integrity and reducing brain edema after neonatal hypoxic-ischemic injury (Chen et al., 2009). In addition, IgG accumulation in the brain is reduced in neonatal MMP-9 knockout (KO) mice compared to wild-type mice 24 h after hypoxia-ischemia (Svedin et al., 2007; Vexler and Yenari, 2009), suggesting that MMP-9 contributes to the opening of the BBB shortly after hypoxia-ischemia. However, there are still several points that need to be clarified. Like many biological mediators, MMPs can be used for good or bad, and this seems to be the case in the brain too. In fact, although MMPs seem to be involved in damage early after stroke, other studies demonstrate that they take on another role later in the process, contributing to repair (Stolp et al., 2005). However, it has been noted that exogenous MMPs kill neurons in culture (Stonestreet et al., 2006; Stolp et al., 2011). This could be due to temporal variations in the action of MMP-9 and/or to the divergent functions of different subtypes of MMPs. These aspects are well reviewed in Lakhan et al. (2013). Briefly, whereas MMP-2 could play a role in the initial opening of the BBB, MMP-9 seems to be more important for the secondary, delayed, opening of the BBB after ischemia, which is necessary for vascular angiogenesis and neuronal regeneration.

A possible explanation for the action of the MMPs is mediation by caveolin-1 (Cav-1). ROS can stimulate MMP activation through the loss of caveolin-1, a protein encoded by the *cav1* gene and a critical determinant of BBB permeability (Gu et al., 2011). Recent studies have revealed that Cav-1 can prevent the degradation of TJ proteins and protect BBB integrity by inhibiting the production of reactive nitrogen species (RNS) and MMP activity. In addition, Cav-1 KO mice show higher rates of apoptotic cell death and larger infarct volumes than wild- in ischemic brains, and the production of NO induces the loss of Cav-1 in focal cerebral ischemia and reperfusion injury. The downregulation of Cav-1 is correlated with an increase in the activity of MMP-2 and -9, decreased ZO-1 expression and increased BBB permeability (Gu et al., 2012).

### Angiogenesis

Although hypoxia-induced angiogenesis is considered beneficial since it allows more oxygen delivery to ischemic regions, there are some associated aspects that are not necessarily positive. One of these aspects is BBB permeability. The main molecules implicated in this process of endothelial cell regression and regrowth are VEGF, placental growth factor, acidic fibroblast growth factor (FGF), TNFα, IL-8 and erythropoietin. The VEGF family has six homologs members, of which the most widely studied is VEGF-A, and 3 tyrosine kinase receptors. Of these, VEGF receptors -1 and -2, which bind VEGF-A, are involved in blood vessel growth, endothelial cell mitogenesis and vasodilatation (via NO-dependent pathways). VEGF is thus a key regulator of vasculogenesis, and endothelial cells that express VEGF also produce MMPs and plasminogen activators to initiate extracellular matrix degradation, the first step in angiogenesis. In response to hypoxia, hypoxia-inducible factor (HIF) -1 is stabilized, leading to the augmented expression of the angiogenic genes mentioned above (Semenza, 2010). Three HIF isoforms are known (HIF-1α, HIF-2α, and HIF-3α), of which HIF-2α is restricted to vascular endothelial cells (Skuli et al., 2009). It has been suggested that in endothelial cells, HIF-1α plays a role in metabolism, proliferation and survival, while HIF-2α is involved in cell migration, adhesion and vascular integrity. Following hypoxia in the neonatal brain, HIF-1α starts to accumulate in neurons, an effect that has been shown to be protective and antiapoptotic (Tahraoui et al., 2001; Tagin et al., 2012). However, in a study evaluating the role of HIF-1α in BBB permeability in a rat subarachnoid hemorrhage model, the elevated expression of HIF-1α in brain tissues temporally coincided with brain edema formation and BBB disruption. In addition, BBB disruption, as determined by the extravasation of Evans Blue (a standard ink tracer used in the CNS), was downregulated by the inhibition of either HIF-1α or MMP-9. In the present study, the inhibition of HIF-1α reduced not only brain edema, but also the expression of aquaporin-4 (see Section on “Edema”) and MMP-9, suggesting that HIF-1α may be a key protein in brain edema formation and BBB disruption, possibly through aquaporin-4 and MMP-9 regulation (Tcharmtchi et al., 2000). Whether the induction of HIF-1α is beneficial or harmful is thus still debatable. Some recent studies have reported that HIF-1α and its target genes may contribute to cell death, tissue destruction and the formation of brain edema, primarily in the acute phase after ischemic brain damage (Thibeault et al., 2001; Thompson et al., 2005). Therefore, inhibiting this molecule immediately following damage could be effective in improving neurological outcome by protecting the brain from secondary injury following edema and BBB disruption (Tcharmtchi et al., 2000).

VEGF also appears to have a dual response to hypoxia-ischemia depending on the time frame studied. Several studies conducted both in adults and, to a lesser extent, in the neonatal brain following hypoxia, show that the i.c. injection of VEGF results in reduced brain injury, with the reduction of brain edema and infarct volume, a decrease in apoptotic cells and BBB permeability (Kaya et al., 2005; Feng et al., 2008). In addition, the inhibition of VEGF receptor-2 decreases endothelial cell proliferation, increases cell death and worsens stroke injury in a neonatal rodent model (Shimotake et al., 2010). The most likely hypothesis is that the “early” upregulation of VEGF (between 1 and 3 h after MCAO) is associated with BBB permeability and contributes to ischemic injury, while conversely, the neuroprotective effects of VEGF, including neovascularization and neuronal protection, occur in the days (48 h) after hypoxia (Marti et al., 2000).

### Edema

One of the most important complications of TBI is edema, which occurs more frequently in the pediatric than in the adult population. This is possibly due to the higher water content of the developing brain and also to the mechanism of regulation of water homeostasis. There are few *in vivo* studies of TBI and BBB disruption in neonates, with most focusing on juvenile rodents. The Badault group has recently evaluated the contribution of the aquaporins to the post-traumatic edema process in juvenile rats and mice (Badaut et al., 2011a,b, 2014; Fukuda et al., 2012, 2013). Pups treated with an inhibitor of aquaporin-4, siGLO siAQP4, show acute improvements in motor function (3 days after injury) and long-term improvements in spatial memory (60 days after injury) when compared with control animals. These improvements are associated with decreased edema formation, increased microglial activation, decreased BBB disruption, and reduced astrogliosis and neuronal death. The effectiveness of the treatment paradigm is associated with a 30% decrease in aquaporin-4 expression at the injection site. Thus, aquaporin's seems to be molecules significantly involved in post-TBI damage, and represent an interesting therapeutic target (Fukuda et al., 2013). Another study has revealed that the inhibition of a kinase involved in the development of brain edema, myosin light-chain kinase, by the administration of an inhibitor, ML-7, to P24 mice starting 4 h after TBI and every 24 h until sacrifice, significantly reduces BBB breakdown and the development of cerebral edema, and preserves neurological function (Rossi et al., 2013).

### Inflammatory response

Neuroinflammation is a key pathological factor in most insults to the developing brain (Degos et al., 2010a). Neuroinflammation is induced by the invasion of pathogens or the release of damage-associated proteins by injured and dying cells. Inflammation includes the activation of microglia, astrocytes and endothelial cells, leading to the secretion of proinflammatory cytokines such as TNFα and IL-1β. There is an increased expression of endothelial adhesion molecules such as vascular cell adhesion molecule 1, intercellular adhesion molecule 1 and the selectins (Simi et al., 2007). Inflammatory processes are, moreover, associated with modifications of the molecular composition or functional state of TJs (Coisne and Engelhardt, 2011). Proinflammatory cytokines also influence the expression of MMPs, which increase BBB permeability by degrading TJs and extracellular matrix components in the endothelial basement membrane (Rosenberg, 2009). These events lead to barrier leakiness, which in turn allows pathogen and immune cell invasion (Labus et al., 2014).

Resident microglia are some of the first cells to respond to inflammatory stimuli. They can migrate, together with astrocytes, to the region of injury or infection, where they clean up cellular debris and produce proinflammatory cytokines such as IL-1β and IL-6. In the case of TBI and hypoxia-ischemia, mast cells also produce large quantities of proinflammatory mediators (Biran et al., 2008; Jin et al., 2009). With regard to the BBB, these cytokines lead to increased permeability and facilitate the entry of peripheral macrophages and cytokines from the systemic circulation. In the instance of inflammation following hypoxic-ischemic injury, this has the effect of exacerbating the excitotoxic cascade by further stimulating glutamate release and free radical and NO production (McLean and Ferriero, 2004). The importance of the mast cell response in causing brain damage after TBI is supported by post-insult treatment with cromoglycate, a mast cell stabilizer that inhibits mast cell degranulation and decreases BBB opening (Strbian et al., 2006), glial activation and neuronal death (Jin et al., 2007) sufficiently to provide long-term neuroprotection.

Leukocyte migration depends on strict interactions with the vascular endothelium, mediated by 3 groups of adhesion molecules: the selectins, the immunoglobulin superfamily (such as intercellular adhesion molecule 1) and the integrins. The pattern of recruitment of monocytes and macrophages is cytokine-specific: IL-1β leads to neutrophil recruitment while TNFα attracts monocytes (Blond et al., 2002). A comparative study in P7 and adult rats subjected to transient middle cerebral artery occlusion shows that Evans Blue extravasation, a measure of paracellular diffusion, the main method of entry for bulky proteins and leukocytes into the brain, remains low during the 24 h period following reperfusion in neonatal rats but is profoundly increased in adult rats. The largely unaltered paracellular diffusion in neonatal rats is associated with the increased expression of several TJ proteins (Vexler and Yenari, 2009) rather than with the decrease in their expression seen in adult rats after a similar injury. In contrast to stroke in adults, in P7 hypoxic-ischemic brains, the transmigration of neutrophils is far lower (Hudome et al., 1997). This may be related to the greater resistance of the BBB to hypoxia-ischemia-induced leaks at this age but also to the immaturity of both endothelial cells, which express less P-selectin (Lorant et al., 1999), and neutrophils, which are less capable of adhering to P-selectin (Tcharmtchi et al., 2000). The lower extent of leukocyte extravasation after ischemic injury in neonatal rats may contribute to the age difference in the structural and functional changes to the BBB after ischemia.

Specific studies have been carried out to examine the effects of inflammation on the functions of the developing BBB and how this influences the development of brain injury. A rather complex and unexpected effect of inflammatory stimuli on BBB integrity and leukocyte transmigration has been reported during the first 3 weeks of life in rats. A comparison of the extent of IgG infiltration in different brain regions following intrastriatal injections of IL-1 or TNFα in rats of different ages shows dramatically higher IgG accumulation in 21-day-old than in 2-h-old rat pups (Anthony et al., 1997, 1998; Schnell et al., 1999). These data demonstrate that at birth, the BBB is functional, and is in fact more resistant to inflammatory stress than in the juvenile brain.

Stolp et al. have conducted two studies of BBB behavior in neonatal, juvenile and adult rats in response to prolonged inflammatory stimuli, in particular the i.p. injection of 0.2 mg/kg lipopolysaccharide (LPS). They have demonstrated for the first time a possible link between changes in BBB permeability (evaluated by permeability to sucrose, inulin and plasma proteins and Cld-5 distribution) and behavioral alterations in animals exposed to the inflammatory stimulus early in development, i.e., on postnatal day (P) 0, P2, P4, P6, and P8. LPS treatment resulted in increased permeability only in adulthood, preceded by Cld-5 alterations in a few vessels at an earlier time point. However, due to the normally low permeability of cerebral blood vessels, it is probable that even a few vessels with altered permeability could significantly contribute to the overall properties of the BBB, and that these changes could explain the short-term changes seen in behavioral tests, in particular in the prepulse inhibition paradigm, that were distinguishable in juvenile animals but not in adult animals. Long-term changes in permeability, as shown by the sucrose permeability test, are correlated in adult rats with altered responses to the dark/light test, suggesting that the impact of inflammation could occur in several phases (short- and long-term) and that each phase could lead to different behavioral modifications (Stolp et al., 2005, 2011). A third study from the same group analyzes, in a similar model (using the opossum *Monodelphis domestica*), the ability of minocycline, a potent anti-inflammatory molecule, to modulate the inflammation-induced changes in BBB permeability and white matter damage following acute and prolonged inflammation during development. The effects of minocycline on inflammation were evaluated in terms of IL-1β mRNA levels in the spleen and brain, and of white blood cell counts in animals sacrificed 1.5 h after the first or fifth LPS injection (single or prolonged inflammatory stimuli). No differences in IL-1β levels were seen between groups, although white blood cells were significantly augmented in animals given prolonged LPS stimulation compared to non-treated animals. Interestingly minocycline significantly reduced this augmentation. The same results were obtained if permeability to ^14^C-sucrose and plasma proteins were evaluated: animals that received a single injection of either LPS or LPS+minocycline showed no changes in BBB permeability to ^14^C-sucrose or plasma proteins. Thus, the inclusion of minocycline probably did not prevent barrier permeability changes after a single LPS injection but prevented barrier permeability to proteins and ^14^C-sucrose when administered during prolonged inflammation.

We have carried out further studies of the BBB in a model in which inflammation alone induces WMD, by the twice-daily i.p. injection of newborn rats or mice with IL-1 over 5 days (Favrais et al., 2011). We evaluated modifications in the expression of the most important choroid plexus TJ proteins at P2, the second day of injections, and 5 days after the beginning of the injections (Favrais et al., 2011; Riddle et al., 2011; Schang et al., 2014). Our results demonstrate that there is no significant non-specific disruption of BCSFB integrity, as assessed by paracellular permeability to tracers, and only a modest increase in Cld-3 expression in the choroidal tissue. In adult animal models of inflammation, the choroid plexus is known to be extremely sensitive to peripheral inflammatory stimuli, as demonstrated by the augmentation of cytokines and other inflammatory markers (Quan et al., 1999; Thibeault et al., 2001; Szmydynger-Chodobska et al., 2012), and the choroid plexus-CSF system appears to be crucial for immune cell trafficking into the CNS during neuroimmune surveillance as well as in the early stages of neuroinflammatory diseases (Reboldi et al., 2009; Schmitt et al., 2012). Thus, studies on the choroid plexus conducted in the context of moderate systemic perinatal inflammation occurring during a period approximating 28–35 weeks of gestation in humans, which alters the developmental program of the white matter, should focus more on the perturbation of specialized functions of the BCSFB, such as neuroimmune regulation, rather than on gross non-specific alterations of barrier integrity.

## Conclusion

Inflammation, vascular reactivity and excitotoxicity are the main protagonists of perinatal diseases, and the BBB and NVU are certainly majorly implicated in these processes. As our knowledge of these processes increases, promising new targets for neuroprotection have been found. However, a lot of questions remain open, especially regarding differences between the behavior of young and adult brains. In addition, even though clinical studies are impossible for obvious ethical reasons, the use of animal models and/or *in vitro* studies impose great limitations in this domain, since the therapeutic options validated in these models are not always as effective when applied to humans. Funding research on the BBB, both in the pre-clinical and clinical domains, should be encouraged in order to better understand early cerebral development and develop effective therapeutic strategies.

### Conflict of interest statement

The authors declare that the research was conducted in the absence of any commercial or financial relationships that could be construed as a potential conflict of interest.

## Abbreviations

BBB: blood-brain barrier
CAV: caveolin
Cld5: claudin-5
CNS: central nervous system
GM-IVH: Intraventricular hemorrhage of the germinal matrix
HIE: hypoxic-ischemic encephalopathy
HIF: hypoxia-inducible factor
IL: interleukin
LPS: lipopolysaccharide
MMP: matrix metalloproteinase
NO: nitric oxide
NOS: nitric oxide synthase
NVU: neurovascular unit
Occl: occluding
P: postnatal day
ROS: reactive oxygen species
TBI: traumatic brain injury
TIMP: tissue inhibitor of metalloproteinases
TJ: tight junction
TNF: tumor necrosis factor
VEGF: vascular endothelial growth factor
WMD: white-matter damage
ZO: zonula occludens.
